# Retraction: Optimized Cu-doping in ZnO electro-spun nanofibers for enhanced photovoltaic performance in perovskite solar cells and photocatalytic dye degradation

**DOI:** 10.1039/d5ra90102b

**Published:** 2025-09-08

**Authors:** Kang Hoon Lee, Rabeea Farheen, Zafar Arshad, Mumtaz Ali, Hamza Hassan, Mubark Alshareef, A. Dahshan, Usama Khalid

**Affiliations:** a Department of Energy and Environment Engineering, Catholic University Korea; b Department of Physics, Government College Women University Faisalabad Pakistan; c School of Engineering and Technology, National Textile University 37640 Faisalabad Pakistan zafarnubii@gmail.com; d Department of Chemical Engineering, University of Engineering and Technology Peshawar Pakistan; e Department of Chemistry, Faculty of Applied Science, Umm Al Qura University Makkah 24230 Saudi Arabia mmshreef@uqu.edu.sa; f Department of Physics, College of Science, King Khalid University Abha Saudi Arabia; g Department of Organic and Nano Engineering, Hanyang University 222 Wangsimni-ro, Seongdong-gu Seoul 04763 Republic of Korea

## Abstract

Retraction of “Optimized Cu-doping in ZnO electro-spun nanofibers for enhanced photovoltaic performance in perovskite solar cells and photocatalytic dye degradation” by Kang Hoon Lee *et al.*, *RSC Adv.*, 2024, **14**, 15391–15407, https://doi.org/10.1039/D4RA01544D.

The Royal Society of Chemistry, with the agreement of the named author, hereby wholly retracts this *RSC Advances* article due to concerns with the reliability of the data.

The XRD patterns in Fig. 2a were first published in ref. 1 as different compounds. The raw data provided for 1% Cu-ZnO, 2% Cu-ZnO and 3% Cu-ZnO are identical.

The Raman spectra for Fig. 2b were first published in ref. 1 as different compounds. The raw data provided for ZnO, 2% Cu-ZnO and 3% Cu-ZnO have obvious signs of manipulation.

The FTIR data in Fig. 4a is extremely similar to the FTIR data in Fig. 4b. The data was first published in ref. 1 and reported as different compounds. The raw data provided for ZnO, 2% Cu-ZnO and 3% Cu-ZnO are identical.

The SEM data for ZnO in Fig. 3b was first published in ref. 2 as another compound.

There are inconsistencies in the appearance of the Tuac plot in Fig. 5b. The authors have not provided raw data and therefore it is not possible to verify the authenticity of the plot.

Given the significance of the concerns with the integrity of the data in the published article and the raw data provided by the authors, the findings presented in this paper are no longer reliable.

This retraction supersedes the information provided in the Expression of concern related to this article.

The authors were informed about the retraction of the article. Zafar Arshad has not agreed with the decision, Mumtaz Ali agreed with the decision, the other authors have not responded.

Signed: Mumtaz Ali

Date: 1st August 2025

Retraction endorsed by Laura Fisher, Executive Editor, *RSC Advances*
